# MicroRNAs in Vertebrate Synapse Development

**DOI:** 10.1100/tsw.2007.196

**Published:** 2007-11-02

**Authors:** Roberto Fiore, Gerhard Schratt

**Affiliations:** ^1^ University of Heidelberg, Interdisciplinary Centre for Neurosciences (IZN), SFB488 Junior Group, Germany; ^2^ University Hospital of Heidelberg, Institute for Neuroanatomy, Im Neuenheimer Feld 345, 69120 Heidelberg, Germany

**Keywords:** microRNA, synapse, local translation, BDNF, dendritic spine

## Abstract

MicroRNAs are a relatively new class of small noncoding RNAs that play an important role in post-transcriptional gene regulation during development and disease. MicroRNAs are abundant in the vertebrate nervous system where they appear to function during neuronal fate determination and early differentiation. It is now becoming increasingly clear that microRNAs are also involved in later stages of neuronal development, namely, the formation and plasticity of synapses. Furthermore, first examples are emerging that microRNAs might contribute to the etiology of neuronal diseases characterized by synaptic dysfunction. This review will summarize the recent examples that describe a function of microRNAs in synapse formation, plasticity, and disease, and discuss future directions that promise to shed light on microRNA regulation by synaptic activity and microRNA function in higher cognitive functions, such as learning and memory.

## THE DEVELOPMENT OF VERTEBRATE SYNAPSES

The precise spatial and temporal formation of synaptic contacts between billions of individual neurons is a prerequisite for the proper functioning of the vertebrate central nervous system (CNS). Synapses that are formed throughout the development of the organism form the basis for cognitive abilities, such as learning and memory. This concept is highlighted by the plethora of neurological disorders that are at least partially caused by synaptic dysfunction, including several forms of mental retardation, epilepsy, and autism.

Synaptic development can be roughly subdivided into two major phases:

1. Synapse formation, which involves guidance of the axon to its prospective partner neuron, and the initial contact between the presynaptic axon terminal and the postsynaptic dendrite. In the case of excitatory synapses, this contact mostly occurs on specialized dendritic protrusions known as dendritic spines. Importantly, synapse formation per se appears to be independent of neuronal activity.

2. Synapse maturation, which involves morphological and molecular remodeling of the pre- and postsynaptic specializations. In contrast to synapse formation, synapse maturation is highly influenced by neuronal activity.

The molecular mechanisms of synapse formation are presented in much detail in a number of excellent reviews[1,2] and will, therefore, not be further discussed here due to space constraints. In the following paragraph, we will give a brief overview of the molecular pathways that contribute in an important way to the activity-dependent maturation, remodeling, and refinement of synaptic connections. Although these mechanisms are discussed here in the context of synapse maturation during development, the very same mechanisms are often involved in the regulation of synaptic plasticity in the adult[3,4,5]. Nevertheless, care should be taken when extrapolating data from developing neuronal cultures to synaptic plasticity in the context of a living animal.

The initial trigger of activity-dependent changes during synapse maturation is the presynaptic release of neurotransmitter. The most obvious consequence is postsynaptic calcium influx, which will induce a number of downstream events discussed below. In addition, activity-dependent release of neurotrophins, such as brain-derived neurotrophic factor (BDNF), also plays a major role.

The molecular mechanism of activity dependent synapse maturation has been a field of intense research during the last 15 years. A number of groups have clearly demonstrated an outstanding role for the trafficking of proteins of the postsynaptic density in activity-dependent synapse maturation[6,7]. These proteins, among others, include members of the AMPA- and NMDA-type glutamate receptor families, numerous scaffolding proteins that anchor receptors to the postsynaptic side, as well as signaling proteins, such as calcium calmodulin-dependent protein kinase II (CamKII). Differential localization of these proteins can be regulated by post-translational modifications, e.g., phosphorylation and palmitoylation[8,9,10]. Concomitantly with protein trafficking, an activity-dependent remodeling of the actin cytoskeleton occurs[11,12]. A major pathway regulating actin dynamics in dendrites and spines involves members of the Rho family of GTPases, Lim-domain containing protein kinase (Limk), and its substrate actin depolymerzing factor (ADF)/cofilin[13]. In addition, ubiquitination and subsequent proteasome-mediated degradation of synaptic proteins has been implicated in activity-dependent control of synapse morphology[5,14,15]. Ubiquitinated proteins include postsynaptic density proteins and, possibly, signaling proteins. Last but not least, proteins synthesized from pre-existing mRNAs in response to neuronal activity are used by synapses to remodel structure and function, notably of dendritic spines, thereby altering synaptic strength in a bidirectional fashion[3,16,17,18]. A peculiarity of protein synthesis in neurons is that it not only occurs in the cell soma, but also in the synaptodendritic compartment close to or within dendritic spines[19]. This so-called local mRNA translation is limited to a specific set of mRNAs that are transported into dendrites in a translationally inactive state by virtue of large ribonucleoprotein complexes (RNPs) that bind to the untranslated regions of the mRNA[20]. This set of mRNAs contains a number of important synaptic proteins, such as CamKIIa, Arc, GluR1/2, Homer, and Limk1[21,22]. The local synthesis of these synaptic proteins is thought to contribute in an important way to synapse remodeling. However, an experiment that unequivocally demonstrates that the local, rather than the somatic, translation of a specific mRNA is required for synapse remodeling is still missing.

On top of the above-mentioned local mechanisms, neuronal activity further induces global changes in mRNA transcription that help to sustain synaptic changes that were elicited locally[23]. A prominent example is the activity-dependent induction of BDNF transcription that creates a positive feedback mechanism that might be important during synaptic growth and maturation.

Despite the wealth of knowledge about molecular mechanisms at the synapse, the inter-relationship among them remains less clear. For example, how is activity-dependent transcription of dendritic mRNAs coupled to the local translation at individual synapses? Similarly, how is regulated turnover of synaptic proteins coupled to their local buildup? Answering these questions will undoubtedly enrich our understanding of synapse-specific, bidirectional changes that occur during development and plasticity.

## MICRORNAS AS POST-TRANSCRIPTIONAL REGULATORS OF GENE EXPRESSION IN NEURONS

The recent discovery of microRNA genes has greatly expanded the cellular toolbox of post-transcriptional regulatory mechanisms. microRNAs are small noncoding transcripts of about 21 nucleotides that preferentially bind to the 3'-UTRs of messenger RNAs (mRNA), thereby either inducing translational repression or degradation of the target mRNA (Fig. 1). MicroRNAs are initially derived from RNA polymerase II transcripts, which are subsequently processed by the RNAse III enzyme Drosha into a precursor microRNA of about 70 nucleotides that is exported to the cytoplasm[24,25,26]. This pre-microRNA is then further cleaved by the cytoplasmic RNAse Dicer to an imperfect, siRNA-like, duplex[27,28]. After unwinding of the duplex, the strand with the lowest pairing energy at the 5' end is preferentially loaded into the RNA-induced silencing complex (RISC), the molecular effector of microRNA and siRNA function[29]. So far, microRNAs have been shown to function in a variety of cellular processes, including developmental timing, apoptosis, differentiation, myogenesis, and glucose homeostasis[30]. Based on bioinformatics prediction, it has been estimated that more than one-third of the human mRNAs could be under the control of the several hundred microRNAs identified so far[31]. Thus, taking into account that the brain displays arguably one of the most complex mRNA expression patterns, it is not surprising that the microRNA pathway appears to fulfill important functions in virtually all facets of nervous system development and function.

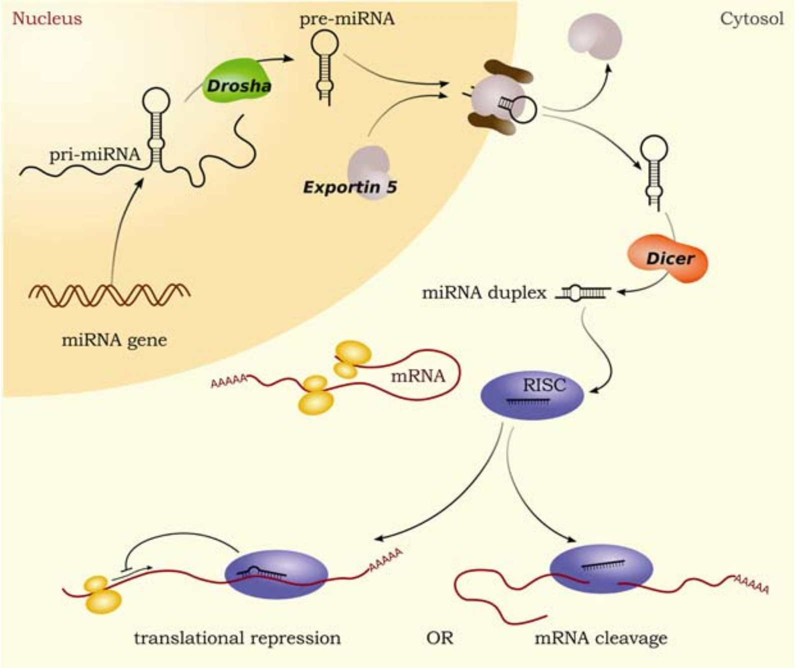

**FIGURE 1.** microRNA biogenesis and modes of action. For details see text.

First evidence for a functional role of microRNAs in the nervous systems came from genetic approaches. In the nematode *C. elegans*, two microRNAs, lsy-6 and miR-273, are required for the asymmetric specification of chemosensory neurons[32]. In *Drosophila*, miR-9a ensures the specification of sensory organ precursors[33], whereas miR-7 is involved in photoreceptor differentiation in the eye[34]. Finally, the complete lack of microRNAs in zebrafish deficient for the processing enzyme Dicer leads to severe defects in brain morphogenesis[35,36].

Based on cloning and expression profiling approaches, microRNAs are also abundant throughout nervous system development of higher vertebrates. Here, very much like in lower organisms, microRNAs appear to have a prominent role in early stages of neuronal fate determination and differentiation[36,37]. The best-studied example in this regard is miR-124, which is required for neuronal differentiation *in vitro* and *in vivo*[38,39,40]. Mechanistically, miR-124 down-regulates a large number of non-neuronal transcripts, thereby helping to establish neuronal cell identity[41].

In the following, we will discuss the emerging role of microRNAs in postmitotic neurons in more detail, providing examples for microRNA function in neuritic outgrowth, synapse maturation, and plasticity.

## MICRORNAS FUNCTION IN SYNAPTIC DEVELOPMENT

In analogy to neuronal development, the first indication that microRNAs are at work in postmitotic neurons to control synaptic development stems from invertebrate model systems. Both biochemical and genetic experiments in *D. melanogaster* show an interaction between components of the microRNA pathway (Dicer, Argonaute) and a regulator of synaptogenesis, the Fragile-X-mental retardation protein (FMRP)[42,43]. Interestingly, FMRP associates with Dicer-derived microRNAs in dendritic processing bodies (P-bodies), suggesting an involvement of microribonucleoprotein complexes (miRNPs) in local control of mRNA translation and stability[44]. The significance of the microRNA pathway for local translational control and plasticity is further supported by the finding that components of the RISC (Armitage, Dicer) are required for a form of long-term memory in *Drosophila*[45]. Regulated degradation of these components correlates with the formation of a stable memory. However, this study did not demonstrate a direct involvement of specific microRNAs in local mRNA translation and long-term memory formation.

In mammalian neurons, work from the Goodman lab provided the first indication that microRNAs function in activity-dependent synaptogenesis. The authors identified the miR-132 microRNA as a target of cAMP response element binding protein (CREB), a transcription factor that is activated in response to neuronal activity and regulates important aspects of neuronal function[46]. The activation of miR-132 transcription correlated with a requirement of miR-132 in neurite outgrowth, a process preceding synaptogenesis. An important downstream target of miR-132 appears to be RhoGAP, a regulator of the neuritic cytoskeleton. However, the implications of these findings for activity-dependent synaptic development await future experimentation.

The growing body of evidence for an important role of dendritic protein synthesis in mammalian neurons prompted us to investigate whether microRNAs could be involved in this process. microRNAs appeared to be ideally suited for regulatory molecules that might participate in the local control of protein synthesis. First, microRNAs in the vertebrate system predominantly inhibit productive translation of mRNAs, a function that could be used to keep dendritic mRNAs dormant during transport and storage near synapses. Second, microRNAs bind to highly specific sequence elements in untranslated regions of mRNAs, potentially allowing them to regulate subsets of mRNAs selectively, e.g., dendritic mRNAs. Third, microRNAs in mature neurons display a high degree of diversity (current estimates speak of about 100 microRNAs in postmitotic neurons), raising the possibility that a microRNA network could coordinately fine tune dendritic protein levels in response to different patterns of synaptic stimulation.

Using sequence inspection of a group of mRNAs responsive to BDNF stimulation, we were able to identify a specific microRNA, miR-134, that was able to bind to the dendritic Limk1 mRNA[47]. miR-134 and Limk1 are found colocalized in dendrites of hippocampal neurons, suggesting that miR-134 and Limk1 interact in neuronal dendrites *in vivo*. miR-134 inhibits the local translation of the Limk1 mRNA, presumably by recruiting RISC. A hallmark of the regulation of dendritic protein synthesis is that it is highly dynamic, meaning that it can switch between translational inhibition and activation depending on the activity status of any given synapse. In terms of the microRNA-mediated inhibition, this would require a relief of the miR-134-imposed inhibition on Limk1 mRNA translation on synaptic stimulation.

Indeed, addition of BDNF, which is released on synaptic activation, counteracted the inhibitory effect of miR-134. This finding suggested a completely novel mode of microRNA control of gene expression. In later studies, microRNA-dependent inhibition of translation was shown to be reversible in other systems, for example, when cells are subjected to stress or during olfactory learning in the fruitfly[45,48].

What are the functional consequences of microRNA function at the synapse? Our studies initially focused on the morphology of dendritic spines, the major sites of excitatory synaptic contact. Since spine size is a good correlate of synaptic strength, it is very often used to infer any alterations in synaptic transmission or plasticity in response to a given treatment[49]. We found that miR-134 negatively regulated dendritic spine size, and that this effect was at least partially mediated by the blockade of Limk1 synthesis in dendrites by miR-134. Together, these results provided the first example of a microRNA that regulates aspects of synapse development and plasticity. Our current model of how microRNAs such as miR-134 function at the synapse (Fig. 2) further sets the framework for several new lines of investigations, some of which we will discuss in the last section.

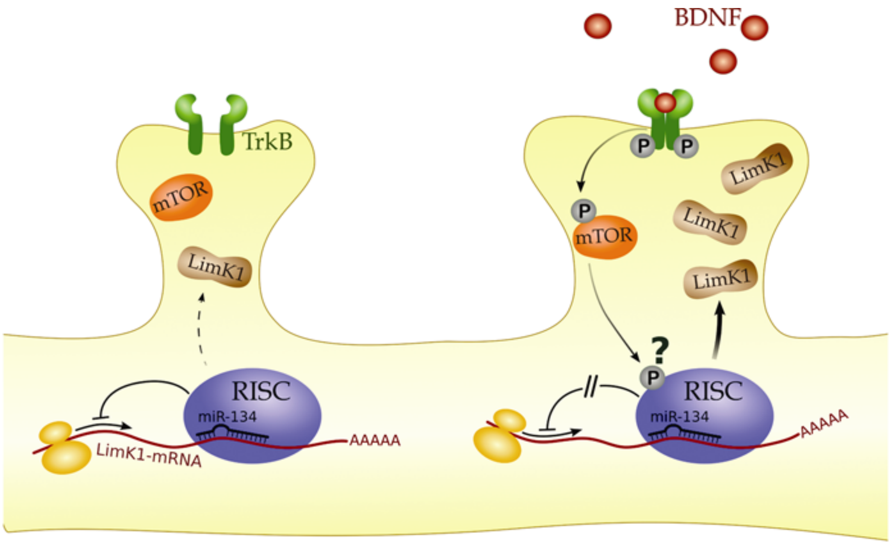

**FIGURE 2.** Working model for the role of microRNAs in dendritic protein synthesis and spine morphology. Left: Dendritic microRNA (i.e., miR-134) recruits the RISC to the dendritic target mRNA (i.e., Limk1), thereby inhibiting mRNA translation and restricting dendritic spine growth. Right: Synaptic stimulation triggers the release of BDNF, whose binding to the Tyrosine-kinase B (TrkB) receptor activates the downstream mammalian target of rapamycin (mTOR) kinase. This leads to an activation of Limk1 mRNA translation, presumably via post-translational modification (i.e., phosphorylation) and inactivation of inhibitory RISC components. Enhanced synthesis of microRNA targets results in dendritic spine growth.

## MICRORNAS IN NEUROLOGICAL DISEASES RELATED TO SYNAPTIC DYSFUNCTION

Based on the above-presented growing evidence for physiological roles of microRNAs in synaptic development, a role for microRNAs in neurological diseases is not at all unexpected. Alterations in spine morphology, e.g., those observed on miR-134 perturbation, are characteristic of many forms of mental retardation, autism-spectrum disorders, Alzheimer's, etc. (for example, spine morphology in patients with Fragile-X mental retardation syndrome (FXS) is not dissimilar from the spine structure observed on miR-134 overexpression or in neurons deficient for the miR-134 target Limk1[50]). Together with the already mentioned genetic interaction between the protein mutated in FXS, FMRP, and components of the microRNA machinery, this strongly suggests that cognitive deficits in FXS patients could at least, in part, stem from perturbed translation of microRNA targets. In this regard, it is rather striking that several identified and predicted targets of synaptic microRNAs have been linked to cognitive disorders (our unpublished observation). For example, a genetic lesion that includes the only bona fide miR-134 target so far, Limk1, causes William Syndrome, a disorder that is characterized, among other things, by defects in visuospatial cognition[51].

Linkage analysis of disease-associated single-nucleotide polymorphisms (SNPs) to microRNA genes promises to reveal yet more links between microRNAs and neurological diseases. In a bioinformatic study that combined SNP genotype data with population genetic techniques, it has been shown that there is a strong negative selection in humans for mutations in microRNA target sites in the 3'UTR[52]. This implies that polymorphisms in predicted microRNA binding sites are likely to be deleterious and, thus, strong candidate regions that might be altered in cognitive diseases. For example, a deleterious polymorphism associated with a microRNA target has been recently discovered in a candidate gene for Tourette's syndrome, SLIT, and Trk-like 1 (SLITRK1). This gene is involved in the regulation of neuronal morphogenesis, and two cases of identical variants in the mir-189 binding site within the SLITRK1 3' UTR have been specifically detected among a population of unrelated individuals with Tourette's syndrome[53]. However, due to the relatively low number of patients used for this study, the significance of these findings for the etiology of Tourette’s syndrome remain uncertain.

Another example of a neurological disease linked to microRNA regulation is spinal muscular atrophy (SMA), a pathology that arises from impaired RNA metabolism. SMA is among the primary genetic causes of infant death, and the disease is caused by progressive degeneration of motor neurons. The responsible gene defect lies within the gene encoding for the RNA-binding protein small motor neuron protein (SMN). This protein is rather ubiquitously expressed and seems to play a variety of roles, ranging from mRNA splicing to axonal transport and local mRNA translation in axons[54]. It is still a conundrum that mutations in a ubiquitously expressed gene can lead to the rather specific degeneration of motor neurons. One hypothesis is based on the finding that several small noncoding RNAs, including microRNAs, are associated with the SMN multiprotein complex[55]. Therefore, microRNAs might be involved in the splicing, trafficking, and/or translation of cargo mRNA that are associated specifically with the SMN complex in motor neurons. Future studies are needed to address whether microRNAs are necessary for SMN-dependent activities, such as mRNA splicing, mRNA transport, and axonal protein synthesis in motor neurons.

## ONGOING WORK AND FUTURE PERSPECTIVES

An obvious unresolved issue at this point is how miR-134 and potentially other dendritic microRNAs enter the synaptodendritic compartment. In principle, such a tiny molecule could travel by simple diffusion, but since a number of neuronal microRNAs are excluded from dendrites (unpublished observation), this possibility appears unlikely. We consider basically two alternative models instead (Fig. 3):

1. “Hitchhiking model”: miR-134 (and possibly other dendritic microRNAs) is processed to its mature, 21-nt form in the neuronal soma and there, together with a number of transport and regulatory proteins, binds to the UTR of mRNAs destined for dendritic transport. At the same time, transport proteins bind to “zip codes” in the mRNA and promote the delivery of the resulting miRNP into dendrites.

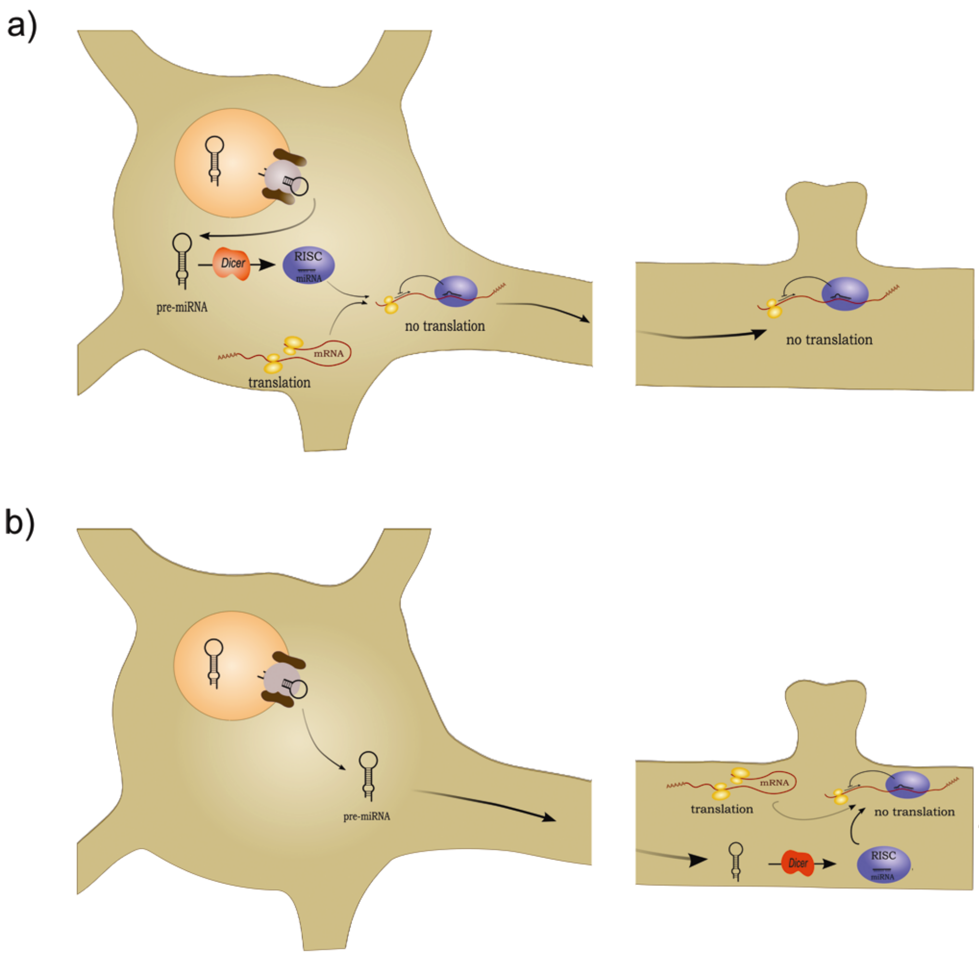

**FIGURE 3.** Possible mechanisms for dendritic transport of microRNAs. (a) "Hitchhiking mode": mature microRNA bind to the target mRNA in the neuronal cell soma, and the microRNA/mRNA complex is actively transported into the dendrites. (b) "Zip code model": dendritic microRNA precursor contains targeting element ("zip code"). This element is recognized by transport proteins that deliver the precursor into the dendrites. For details see text.

2. "Zip code model": the miR-134 precursor (or even the pri-miR-134) contain their own "zip codes" that are recognized by the transport proteins. In this model, processing of the precursor microRNA is assumed to occur locally in the synaptodendritic compartment, a scenario that would add another level of regulation to microRNA function.

Consistent with the latter model, enzymes of the microRNA processing machinery (e.g., Dicer, eIF2C2) are found in the dendrites close to synapses[56]. It is important to note here that none of these two models has any experimental support so far.

A focus of our work is to understand how microRNA function is regulated by neuronal activity in the context of activity-dependent processes, such as dendritogenesis and synaptogenesis. An important aspect of this regulation occurs at the level of activity-dependent transcription. Accordingly, we recently found that a large cluster of about 50 neuronal microRNAs, including miR-134, is activated on calcium influx elicited by membrane depolarization (R.F., Khudayberdiev, and G.S., in preparation). This effect appears to be partly mediated by the activity-induced transcription factor MEF2 (myocyte enhancing factor 2), which was recently identified as a negative regulator of synaptogenesis[57]. We are currently exploring whether blocking the function of members of the miR-134 cluster interferes with activity-dependent dendritogenesis/synaptogenesis. Another potential implication of this study is that large groups of coregulated microRNAs could operate in a combinatorial fashion, presumably on common target mRNAs, to shape gene expression in different cellular locales in response to extracellular stimulation. Such a "microRNA code" could be important in neurons to adjust gene expression profiles to different patterns of synaptic stimulation.

In addition to global changes in gene transcription, neuronal activity also affects local mRNA translation in dendrites. Based on our previous work, this also appears to apply to microRNA-mediated inhibition of local protein synthesis. We found that the inhibitory function of miR-134 on Limk1 mRNA translation could be relieved by BDNF, a peptide that is synthesized and released by neuronal activity. Currently, we are trying to understand the molecular mechanism of the derepression. Therefore, biochemical purification and functional dissection of the microRNA-associated protein complexes will be needed. The identification of functionally important components of such complexes will be a prerequisite for future experiments concerning activity-induced post-translational modifications and remodeling of microRNA-associated proteins.

Beyond miR-134, it will be interesting to characterize the whole pool of dendritic microRNAs and their target mRNAs, especially in respect to the above-mentioned combinatorial "microRNA code". For this purpose, we are currently applying two different technologies: microarray-based expression profiling and deep sequencing of synaptosomal microRNAs using the recently developed 454 technology. Preliminary results indicate that synaptosomes contain a specific subset of microRNAs, some of which display important roles in dendritic spine morphogenesis (Siegel, R.F., and G.S., in preparation). Gene ontology searches combined with target validation should reveal if these microRNAs regulate overlapping or rather distinct sets of target mRNAs at the synapse.

Finally, investigations on the physiological roles of microRNAs in the vertebrate brain *in vivo* are in the early steps, but promise to give more definitive answers about their significance for cognitive function and behavior. The analysis of mutant mice lacking Dicer, specifically in the postnatal brain, should give a first indication of the relevance of microRNA regulation in postmitotic neurons. In addition, knockout mice for candidate microRNAs that appear to be involved in synapse development are currently generated. An alternative approach consists of the transduction of postmitotic neurons with viruses expressing the microRNA of interest. This can be easily achieved by intraventricular injection of newborn mouse or rat pups with adeno-associated virus (AAV) or lentivirus[58,59]. One major drawback at the moment is that virus-mediated delivery does not allow specific interference with endogenous microRNAs. A future solution to this problem could be, for example, the direct injection of cell permeable antisense microRNA inhibitors, so-called antimirs[60,61]. These molecules have proven effective in inhibiting microRNA function in organs such as the liver once injected into the bloodstream, but usage in the brain will require more sophisticated delivery strategies, i.e., peptide conjugation[62].

## CONCLUDING REMARKS

Although the evidence for microRNA function at the synapse is rather limited at present, it can be anticipated that studies on the hundreds of microRNAs expressed in mature neurons and their respective targets will greatly enrich our understanding of synaptic development and plasticity mechanisms. The ability to develop highly specific inhibitors for each of these molecules further gives hope that they might serve as targets for therapeutical intervention in cognitive disorders in the near future. In this way, microRNAs could literally become molecules to remember.

## ACKNOWLEDGMENTS

We thank G. Siegel and S. Bicker for help with the illustrations. This work was supported by the Deutsche Forschungsgemeinschaft (DFG/SFB488). G.S. is a recipient of the Career Development Award from the Human Frontier Science Program (HFSP).
